# Spatial Analysis of Slowly Oscillating Electric Activity in the Gut of Mice Using Low Impedance Arrayed Microelectrodes

**DOI:** 10.1371/journal.pone.0075235

**Published:** 2013-10-04

**Authors:** Mizuki Taniguchi, Shunichi Kajioka, Habibul B. Shozib, Kenta Sawamura, Shinsuke Nakayama

**Affiliations:** 1 Department of Cell Physiology, Nagoya University Graduate School of Medicine, Nagoya, Japan; 2 Department of Urology, Graduate School of Medical Sciences, Kyushu University, Fukuoka, Japan; Haassah Medical Center, Israel

## Abstract

Smooth and elaborate gut motility is based on cellular cooperation, including smooth muscle, enteric neurons and special interstitial cells acting as pacemaker cells. Therefore, spatial characterization of electric activity in tissues containing these electric excitable cells is required for a precise understanding of gut motility. Furthermore, tools to evaluate spatial electric activity in a small area would be useful for the investigation of model animals. We thus employed a microelectrode array (MEA) system to simultaneously measure a set of 8×8 field potentials in a square area of ∼1 mm^2^. The size of each recording electrode was 50×50 µm^2^, however the surface area was increased by fixing platinum black particles. The impedance of microelectrode was sufficiently low to apply a high-pass filter of 0.1 Hz. Mapping of spectral power, and auto-correlation and cross-correlation parameters characterized the spatial properties of spontaneous electric activity in the ileum of wild-type (WT) and *W/W^v^* mice, the latter serving as a model of impaired network of pacemaking interstitial cells. Namely, electric activities measured varied in both size and cooperativity in *W/W^v^* mice, despite the small area. In the ileum of WT mice, procedures suppressing the excitability of smooth muscle and neurons altered the propagation of spontaneous electric activity, but had little change in the period of oscillations. In conclusion, MEA with low impedance electrodes enables to measure slowly oscillating electric activity, and is useful to evaluate both histological and functional changes in the spatio-temporal property of gut electric activity.

## Introduction

Cellular electrical cooperation produces smooth and elaborate motions of various biological systems. In the gut, it is well known that a network of intrinsic neurones simultaneously induce ascending contraction and descending relaxation of smooth muscle, leading to peristaltic movements [1], [2]. Also, basal slow electric oscillations occur in most sections of the gastrointestinal tract [3], [4].

Relatively recent studies have revealed that special interstitial cells, referred to as interstitial cells of Cajal (ICC) act as pacemaker cells for the basal electric activity [5]–[9]. These cells are likely to contribute to spatial organization of gut excitability through their network of long processes. In agreement with this notion, there is a growing body of evidence that gut motility disorders, such as diabetic gastroparesis and inflammatory bowel diseases (IBD) among other diseases, contain alterations of the network-forming pacemaker cells as well as neurons and smooth muscle cells [10]–[14]. Thus, investigation into the spatial property of electrical activity, including in pacemaker cells, benefits a more precise understanding of gut motility and medical therapy. In addition, interstitial cells mimicking ICC are distributed over the body, for instance in urinary tracts, lymph ducts and small vessels, and are now considered to play a crucial role in generating spontaneous electric activity.

Using an 8×8 microelectrode array (MEA), we previously compared spontaneous basal electrical activity of the ileum between wild-type (WT) and *W/W^v^* mice. In the latter, it is well known that the number of ICC is reduced thereby their pacemaker and network functions are impaired due to a loss-of-function mutation of c-Kit receptor gene [5], [7], [15]. A power spectrum integrating the whole recording area could distinguish these preparations [16] in the presence of nifedipine and tetrodotoxin (TTX), which suppress the electrical activity of neurones and smooth muscle, respectively. Also, potential mapping videos qualitatively suggested the uncoordinated spontaneous electric activity in the ileum of *W/W^v^* mice. However it was preliminary to display the coordinated actions between basal slow electric oscillations over the whole recording area. In this study, we thus analyzed the MEA field potential recordings by using auto-correlation and cross-correlation parameters as well as spectral power. Examples show that mapping analyses could well characterize spatial properties of gut spontaneous electric activity based on both functional and histological alterations. The ICC network appears to play a crucial role in coordinating gut electric activity with a delay of several seconds per millimetre, and requires the support of other cellular components to enhance the coupling. Also, we carefully explain the requirements of MEA systems for the measurement of slowly oscillating electric potentials in a small area, in order to address recent controversies on the frequency of gut spontaneous electric activity [17]–[21].

## Materials and Methods

### Animals and Preparations

Animals were treated ethically, in accordance with the guidelines for proper conduct of animal experiments in Science Council Japan. All procedures were approved by the Animal Care and Use Committee in Nagoya University Graduate School of Medicine (Permission #23357). C57BL/6J (WT) and *W/W^v^* mice (∼8 weeks after birth) were sacrificed by cervical dislocation after deeply anaesthetising with diethyl ether. The ileum was quickly excised, and cut along the mesenterium. The whole-muscle layer (∼5 mm×20 mm) containing the myenteric plexus was isolated using fine forceps. The mice used were purchased from Chubu Kagaku Shizai Co., Ltd. (Nagoya, Japan).

### Electrical Recordings

**Figure 1:**
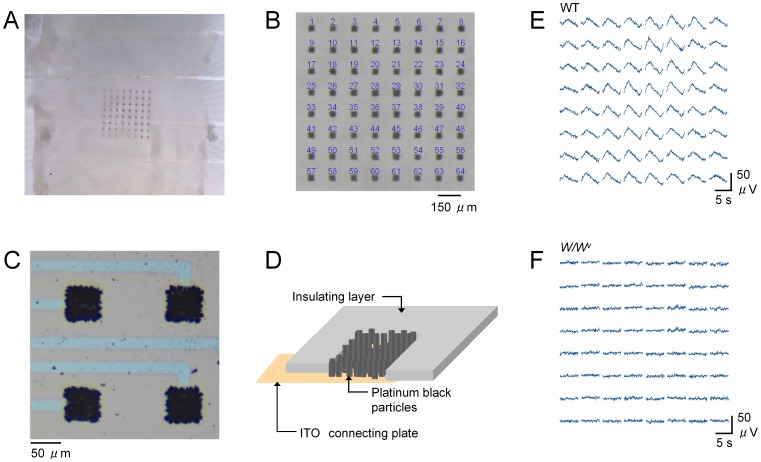
8×8 MEA recording. A) Ileal musculature from mice mounted in a recording chamber with an 8×8 MEA in the centre. Extracellular field potentials were simultaneously measured at 64 channels with a high-pass filtering of 0.1 Hz. B) Alignment of MEA recording channels. C) Recording microelectrodes shown with a large magnification. D) Scheme of a recording electrode. Platinum black particles are fixed on an ITO connecting plate. Electrodes are separated with insulating polymers. E–F) Each set of 64 field potentials represent an example of simultaneous recording from ileal musculatures of WT or *W/W^v^* mice in normal solution. Digital filter was not applied. Traces show field potentials of only 5 s.

To measure ileal electric activity, an array of 8×8 planar microelectrodes with an inter-electrode distance of 150 µm was used (Alpha MED Scientific, Ibaraki, Japan). Ileal musculatures were fixed in a recording chamber with this MEA, with the longitudinal muscle layer placed directly on microelectrodes under the strings of a slice anchor (SDH series, Harvard Apparatus Japan, Tokyo Japan) (Fig. 1A–B). The recording chamber (∼1 ml in volume) was perfused with an extracellular solution at a constant rate of 1–2 ml min^−1^ and placed on a heater kept at ∼34°C. To minimize the electric noise, small dripping tubes for electric isolation were inserted in both the perfusion in-line and out-line.

Each recording electrode is a ∼50 µm square, made by platinum black nanoparticles [22], thereby the surface area is increased by ∼200-folds, being equivalent to 0.5 mm^2^ (Fig. 1C–D; see also Fig. S1). Platinum black particles are fixed on a small square (∼50 µm×50 µm) area of an indium tin oxide (ITO) plate (50 and 500 µm thick, respectively). To insulate the connecting lines (extended ITO plate), the upper surface of the recording chamber is coated with polyimide, leaving the square areas of microelectrodes.

The impedance of the recording microelectrode (*Z*_ME_) is given as a function of signal frequency (*f*):
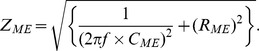



The capacitance (*C*_ME_) and resistance (*R*_ME_) of each microelectrode measured by an LCR meter (ZM2353, NF Corp., Yokohama, Japan) were 0.052 µF and 15 kΩ, respectively. The impedance of the recording electrode at 0.1 Hz was therefore small enough [∼31 MΩ = √ {1/(2π×0.1 Hz×0.052 µF)^2^+ (15 kΩ)^2^}] to follow oscillating potentials, compared to the input impedance of the multi-channel amplifier (100 MΩ at 0.1 Hz).

**Figure 2:**
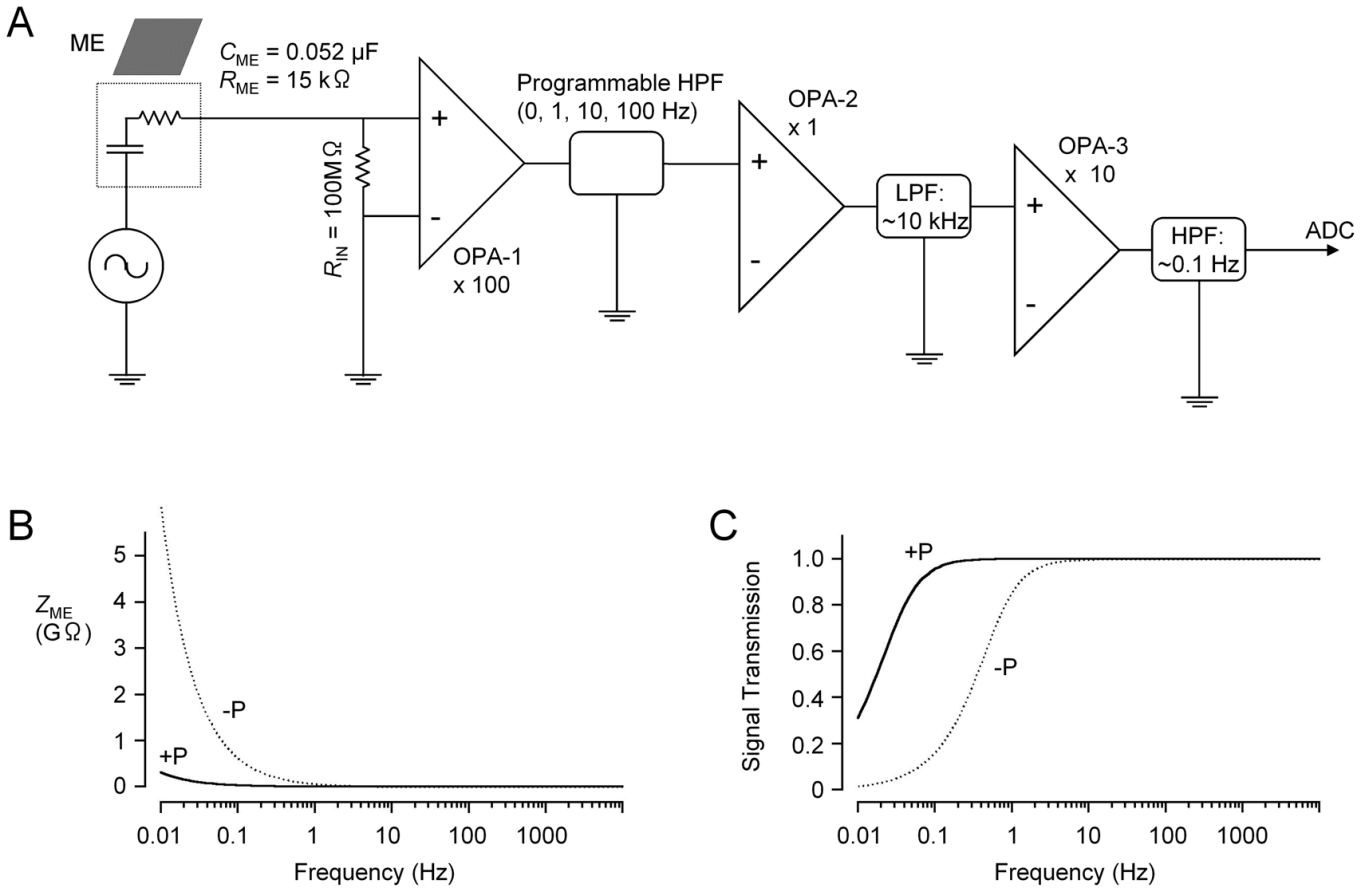
Simplified electric circuit diagram, and simulation of the frequency-dependence of circuit parameters. A) Three operational amplifiers (OPA-1, 2 and 3) are sequentially connected in each recording channel, and electric signals are recorded through an A/D converter (ADC). The input resistance (*R*_IN_) of OPA-1 is set to be 100 MΩ by connecting a resistor to the reference (ground). B–C) The impedance of microelectrode (*Z*_ME_) and the efficiency of signal transmission in OPA-1 are plotted against the frequency. Continuous and dotted lines represent microelectrodes with and without platinum black nanoparticles (+P and –P), respectively. The capacitance and resistance of microelectrodes (*C*_ME_ and *R*_ME_) with platinum black were measured to be 0.052 µF and 15 kΩ, respectively. *C*_ME_ without platinum black was assumed to be smaller by 200-folds.

The efficacy of electric signal transmission (*Tr*) is estimated from the input resistance of the amplifier (*R*_in_) and the impedance of the electrode at a certain frequency:
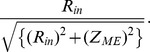



Thus, Tr was estimated to be ∼95% at 0.1 Hz: 100 MΩ/√ {(100 MΩ)^2^+ (31 MΩ)^2^} (Fig. 2).

The low resistance (15 kΩ) of electrodes are advantageous for reducing the thermal noise (*N*_T_):
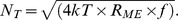
where *k* and *T* are the Boltzmann constant and absolute temperature, respectively. The calculated *N*_T_ was ∼1.6 µV at the low-pass frequency (10 kHz) of the amplifier. Also, in order to reduce the interface tension, recording electrodes were immersed in 0.1% polyethyleneimine overnight prior to the first use, and kept in distilled water.

When the noise level of one recording channel was twice larger than that of other channels, the arrayed data were judged to be inappropriate for 2D pseudo-colour images, described below. Air bubbles on microelectrodes were frequently the cause of large noise levels. When removals of platinum black nanoparticles from microelectrodes (i.e. high resistance and impedance) were the cause, the recording chamber was replaced with new one.

A set of 8×8 field potentials (Fig. 1E–F) were recorded in a personal computer via a computer-controlled, multi-channel AC amplifier with low-pass filtering at 10 kHz, and 14 bit A/D converters with a sampling rate of 20 kHz. The dynamic range of A/D conversion applied was usually ±1 mV. A high-pass filter of 0.1 Hz was applied to stabilize the baseline drift of the microelectrode potential. It is known that such low cut-off frequency of high-pass filtering is necessary to follow slow electric activity in the gut [23], [24].

MEA measurements were started after perfusing the recording chamber with a normal extracellular solution for at least 30 min. In some experiments, nifedipine (1 µM) and TTX (250 nM) were added to suppress smooth muscle and neuronal electric activity [16], [25] and predominantly measure ICC electric activity. Nifedipine also suppressed mechanical activity by blocking L-type Ca^2+^ channels, a major pathway for Ca^2+^ influx in visceral smooth muscle [14].

### Solutions and Drugs

The “normal” extracellular solution, a modified Krebs solution, had the following composition (in mM): NaCl, 125; KCl, 5.9; MgCl_2_ 1.2; CaCl_2_ 2.4; glucose 11; Tris-HEPES, 11.8 (pH 7.4). Nifedipine and TTX were purchased from Sigma-Aldrich (St Louis, MO, USA).

### Analyses and Statistics

**Figure 3:**
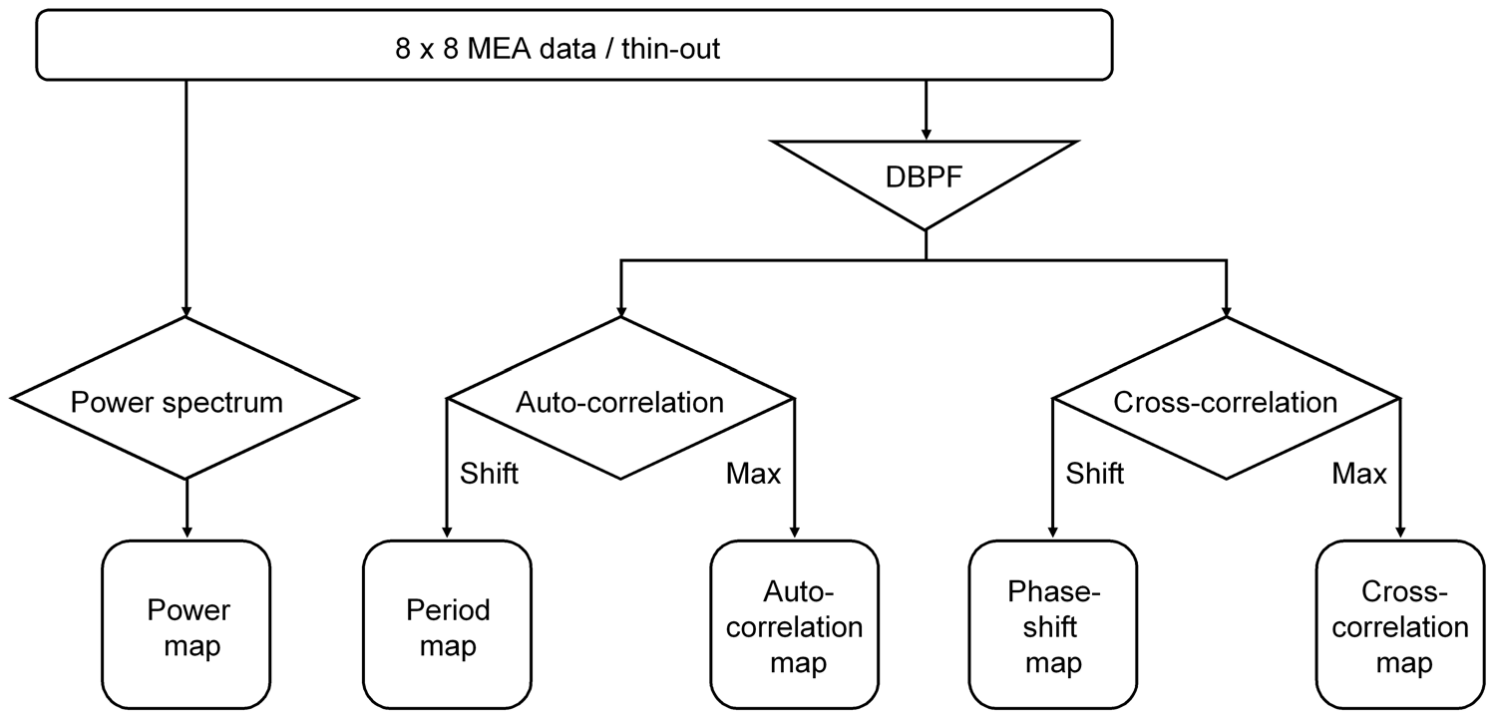
Procedures used in MEA analyses. 8×8 array data of field potentials were normally thinned by a 1000-fold time domain, thereby the sampling interval was increased to 50 ms. 1) In power spectrum analysis, Fourier transformation was applied to each field potential recording (1024 points for ∼52 s), and the spectral power in the frequency between 9.4 to 27.0 cpm was plotted as a 2D pseudo-colour image (Power map). 2) Auto-correlation function was derived from each field potential recording (1024 points) of 8×8 array data, after digital band-pass filtering (DBPF) of 0.05–0.5 Hz. The shift to the next peak was used as a period of spontaneous electric activity. 3) Cross-correlation function was derived from each field potential recording (1024 points) of 8×8 array data using Ch28 as a base channel, after DBPF of 0.05–0.5 Hz. The shifts (time-lags) and cross-correlation values of the peak in cross-correlation curves were plotted as 2D pseudo-colour images (Phase-shift map and Cross-correlation map, respectively).

The procedures for analysing 8×8 MEA data are summarized in Fig. 3. Arrayed data of field potential recordings were thinned by a 1000-fold time domain, thereby the sampling interval was increased to 50 ms. This sampling frequency was enough to follow ICC pacemaker activity. Digital band-pass filter, power spectrum and cross-correlation analyses were performed using commercial add-in software (Kyowa Electronic Instruments, Tokyo, Japan).

**Figure 4:**
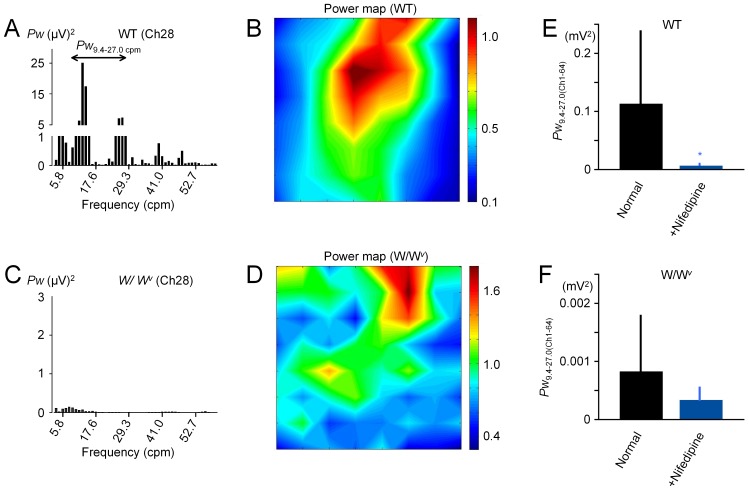
Spectral power analysis. A–B) Power spectrum transformed from field potentials at Ch 28 and pseudo-colour map constructed from the same array data used in Fig. 1E (the ileum of a WT mouse). C–D) Power spectrum and pseudo-colour map for a *W/W^v^* mouse preparation. The data in Fig. 1F was used. Note the spectral peak corresponding to the oscillation frequency seen only in WT mice. E–F) Bar graphs E and F show the effect of nifedipine (1 µM) and TTX (250 nM) on the spectral power summed in all channels in WT and *W/W^v^* mice, respectively. These drugs were applied to record ICC pacemaker activity by suppressing smooth muscle and enteric neurones. Asterisks represent statistical significance (*P*<0.05). Note that the Y-axis is expanded in C and F.

**Figure 5:**
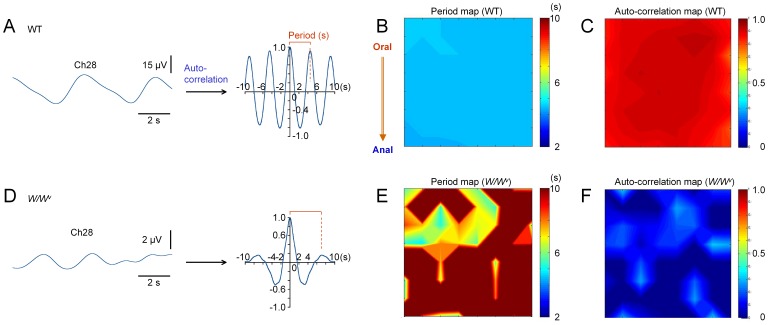
Auto-correlation analyses. Panel A shows an auto-correlation function constructed from the field potential recording at Ch28 in an ileal musculature of WT mice (the same preparation shown in Fig. 1E and Fig. 4A). Panels B and C show the distribution of the period (x-axis) and amplitude (y-axis), respectively, estimated from the adjacent peak of autocorrelation function, in the same ileal preparation shown in A. Panels D–F show auto-correlation function, period map and auto-correlation map, respectively, in the ileal musculature of *W/W^v^* mice (the same preparation in Fig. 1F and Fig. 4D). The period and amplitude of autocorrelation were set to 10 in the period map, and to 0 in the autocorrelation map, respectively, in recording channels in which no peak was observed (the peak was below nought) in the frequency range of 9.4–27.0 cpm.

**Figure 6:**
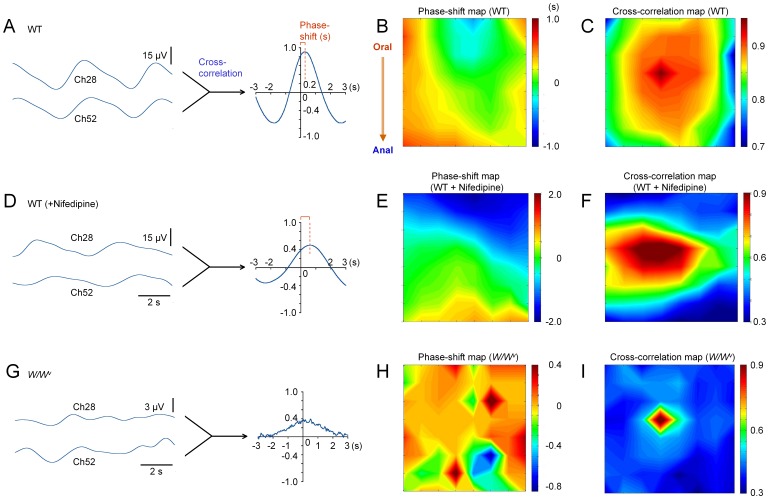
Cross-correlation analyses. Panel A shows a cross-correlation function constructed from field potential recordings at Ch28 and Ch52 in an ileal musculature of WT mice (the same preparation shown in Fig. 1E). Panels B and C show the distribution of the phase-shift (x-axis) and the amplitude (y-axis), respectively, of the peak in cross-correlation functions derived from all 64Ch MEA data, in the same ileal preparation shown in A. Ch28 was used as the base channel. Panels D–F show cross-correlation function, phase-shift map and cross-correlation map, respectively, in the presence of nifedipine and TTX in the same preparation shown in A–C. Panels G–I were constructed from MEA data recorded from an ileal musculature of *W/W^v^* mice (the same preparation in Fig. 5D–F).

1) In power spectrum analysis, Fourier transformation with a Hanning window was applied to each field potential recording (1024 points for ∼52 s) of the 8×8 array data. The spectral power in the frequency range between 9.4 to 27.0 cpm (*Pw*_9.4–27.0_) was plotted as a 2D pseudo-colour image (Power map) (Fig. 4E and F), using MATLAB software. 2) The auto-correlation function was derived from each field potential recording (1024 points) of 8×8 array data, after digital band-pass filtering of 0.05–0.5 Hz. The shift and amplitude of the peak adjacent to the centre peak were plotted as 2D pseudo-colour images: period map (Fig. 5B and E) and auto-correlation map, respectively (Fig. 5C and F). 3) The cross-correlation function was derived from each field potential recording (1024 points) of 8×8 array data using Ch28 (or Ch29) as the base channel, after digital band-pass filtering of 0.05–0.5 Hz. The shift (time-lags) and amplitude of the peak of cross-correlation functions (curves) were plotted as 2D pseudo-colour images: phase-shift map (Fig. 6B, E and H) and cross-correlation map, respectively (Fig. 6C, F and I). In the ileum of in *W/W^v^* mice, no digital band-pass filter was applied to 8×8 array data, because oscillating spontaneous electric activity was so small in this tissue that cross-correlation functions reflected the base-line drift after digital band-pass filtering. Also, it is noted that in auto-correlation analysis and cross-correlation analysis, the band-pass filter of 0.05–0.5 Hz was appropriate to mainly detect a basal oscillation frequency of ICC pacemaker activity, but reduced spike-like transient components in field potential traces.

Numerical data are expressed as means±S.D. Significant differences were evaluate by paired and unpaired *t*-tests.

## Results

### Microelectrode Array Recording of Ileal Pacemaker Activity

Isolated ileal musculatures were mounted in a recording chamber with an 8×8 MEA on the bottom (Fig. 1A), and field potentials were simultaneously recorded through a multi-channel AC amplifier. Microelectrodes were aligned with a distance between them of 150 µm, reflecting ileal pacemaker activity of a ∼1 mm^2^ area (B). Due to the increased surface area of the electrodes made by fixing platinum black particles (Fig. 1C–D), a high-pass filter of 0.1 Hz could be applied in the AC amplifier in order to follow slowly oscillating electric activity. The transmission of the electric signal was ∼95% at 0.1 Hz (see Materials and methods). The panels in Fig. 1E and F show example recordings of 8×8 field potentials from isolated musculatures of WT and *W/W^v^* mice, respectively (n = 8 vs 5), under superfusion with a ‘normal’ extracellular solution. Field potentials were smaller in the latter, agreeing well with previous reports on the impairment of the ICC (i.e. pacemaker cells) network [5], [15]. During prolonged recordings for more than several minutes, slow potentials were continuously observed in WT mice.

### Spectral Power in WT and W/W^v^ Mice

First, spectral analysis was performed to compare these preparations. The panel in Fig. 4A shows the power spectrum transformed from a field potential recording at 28Ch (for ∼52 s: 1024 points) in the same preparation of WT mouse shown in Fig. 1E. Based on the spectral power between 9.4 and 27.0 cpm (*Pw*_9.4–27.0_) covering the ileal pacemaker frequency under normal conditions, the pseudo-colour map in Fig. 4B was constructed by normalizing with *Pw*_9.4–27.0_ at 28Ch, in order to indicate the distribution of the magnitude of spontaneous electric activity. Similarly, the power spectrum (Fig. 4C) and pseudo-colour map (Fig. 4D) were constructed for the ileal musculature preparation of the *W/W^v^* mouse shown in Fig. 1F. The comparison between these preparations indicated that in addition to a large difference of the total spectral power over the recording area, the magnitude of electric activity gradually changed in WT mice (Fig. 4C), while it was rather randomly distributed in *W/W^v^* mice (Fig. 4D), agreeing well with the notion that the ICC network plays an important role in coordinating the local electric activity to produce smooth contraction [16].

Smooth muscle and enteric neurones are major components of electrically excitable cells other than ICC. Nifedipine and TTX suppress these cells by blocking voltage-gated L-type Ca^2+^ channels and Na^+^ channels, respectively [16], [25], thus these drugs can be used to examine how much electric current is generated by ICC. After the application of nifedipine (1 µM) and TTX (250 nM), the sum of *Pw*_9.4–27.0_ in all 64 channels was largely reduced in ileal musculature preparations of WT mice (from 0.11±0.12 to 0.0062±0.0052 mV^2^, *P*<0.05, paired *t*-test, n = 8) (Fig. 4E). On the other hand, *Pw*_9.4–27.0_ was small in ileal musculatures of *W/W^v^* mice even in normal solution. Application of these drugs again reduced *Pw*_9.4–27.0_ in these preparations (from 0.00083±0.00098 to 0.00033±0.00025 mV^2^, *P* = 0.20, paired *t*-test, n = 5) (Fig. 4F), but the ratio of the remaining power was larger in *W/W^v^* mice (5.5% vs 40.3%). The results suggest that ICC facilitated the electric activity of other excitable cells in ileal musculatures.

### Auto-correlation Analysis

Next, auto-correlation analysis was performed to compare these preparations in terms of the frequency of spontaneous electric activity. Panels in Fig. 5A–C show an example of auto-correlation analysis obtained from the ileal musculature of WT mice in normal solution: the array data in Fig. 1E (and Fig. 4B) was used. An auto-correlation function (curve in A) was derived from a field potential recording at Ch 28 (centre channel). Likewise, auto-correlation functions were derived from all MEA channels. The shift toward the adjacent peak (x-axis) was used for mapping the period of electric oscillation in each channel (period map: Fig. 5B), while the amplitude of the adjacent peak (y-axis) was used as an index of similarity of electric oscillations in a time-domain (auto-correlation map: Fig. 5C). The same procedures of auto-correlation analysis were applied to the array data in Fig. 1F (and Fig. 4D) to draw Fig. 4D–F: an auto-correlation function derived from a field potential data at Ch28, period and auto-correlation maps, respectively. The period and auto-correlation maps differed significantly between WT and *W/W^v^* mice.

The periods of oscillations estimated over the recording area were distributed within a small range (4.35–4.5 s in 64 channels: Fig. 5B) in WT mice, but varied largely in *W/W^v^* mice (4.8–8.55 s, in 35 channels: Fig. 5E; Peaks were not detected in 29 out of 64 channels in the frequency range of 9.4–27.0 cpm). Correspondingly, the period map was monotonous in colour in WT mice, but was bumpy in *W/W^v^* mice. The average of the period was 4.2±0.6 s (n = 8) in WT, and 6.1±1.9 s (n = 5) in *W/W^v^* mice. The standard deviation of the mean of the period in 64 channels over the recording area was 0.099±0.059 (n = 8) in WT, and 0.38±0.17 (n = 5) in *W/W^v^* mice, indicating that the frequency estimated were spatially much more varied in *W/W^v^* mice than in WT mice (*P* = 0.02, unpaired *t*-test). These results reinforced that pacemaker cells are impaired in ileal musculature preparations in *W/W^v^* mice.

In addition, the amplitudes of the adjacent peak of auto-correlation functions were much smaller in *W/W^v^* mice (Fig. 5D) than in WT mice (Fig. 5A): 0.41±0.27 (n = 8) in WT mice; 0.14±0.02 (n = 5) in *W/W^v^* mice; *P* = 0.01, unpaired *t*-test. The channels in which the peak of auto-correlation function was below nought in the range of 9.4–27 cpm were not involved in the statistics for the period.

### Cross-correlation Analysis

In order to assess the synchrony of spontaneous electric activity recorded at MEA channels, cross-correlation analysis was performed (Fig. 6). Panels in Fig. 6A–C show an example of such analysis for spontaneous electric activity in the ileal musculature of WT mice in normal solution. The same array data in Fig. 1E and Fig. 5A–C was again used. A cross-correlation function (curve in Fig. 6A) was derived from a field potential recording at Ch52 using a centre channel (Ch28) as the reference. Likewise, cross-correlation functions were derived from all MEA channels, and the shift (x-axis) and amplitude (y-axis) of the peak in each cross-correlation function were plotted to draw a phase-shift map (Fig. 6B) and a cross-correlation map (Fig. 6C), respectively.

Panels in Fig. 6D–F show the cross-correlation analysis applied to the MEA data obtained 20 min after application of nifedipine and TTX in the same ileal musculature preparation of WT mice. On the other hand, panels in Fig. 6G–I show the MEA data of *W/W^v^* mice (in normal solution) used in Fig. 1F. Each set of the cross-correlation analysis consists of cross-correlation-function derived from Ch28 and Ch52, phase-shift and cross-correlation maps, respectively. A comparison of the phase-shift and cross-correlation maps between normal solution (Fig. 6B–C) and in the presence of nifedipine and TTX (Fig. 6E–F) indicated that these drugs reduced the propagation velocity and coupling of spontaneous electric activity along the oral-to-anal plane (longitudinal muscle: vertical plane). Similar inhibitory effects on propagation were observed in 5 out of 8 preparations of WT mice: the phase shift at 52Ch was prolonged from 0.23±0.16 s to 0.59±0.49 s (n = 5). The application of drugs, nevertheless, had little effect on the period of spontaneous electric activity estimated by auto-correlation (4.2±0.6 s vs 4.6±0.6 s, *P*>0.05, paired *t*-test, n = 8). On the other hand, analyses of *W/W^v^* mice indicated that spontaneous electric activities were not correlated even with the support of smooth muscle and neurons. Namely, the phase-shifts between electric activities were randomly distributed (Fig. 6H), and the amplitudes of cross-correlation peaks were low, except in the centre channel used as a base channel in cross-correlation analysis (Fig. 6I). These results indicated that the ICC network plays an essential role in propagating electric activity in ileal musculatures.

## Discussion

To investigate the spatial properties of gut electrical activity, multiple conventional intracellular microelectrodes have previously been applied [26], [27]. Also, due to recent progress, optical probes, including fluorescent proteins, could be applicable in future studies [28], [29]. However, the former technique largely depends on personal skill and is performed in a limited number of laboratories with specialists. Also, the latter requires voltage-sensitive probes with a sufficiently large change in fluorescence, and frequently affects physiological properties of cellular tissues while loading probes. On the other hand, the measurement of MEA is straightforward. After removal of the mucous membrane, musculature preparations are simply mounted on MEA, and MEA measurements on their own never damage preparations. Thus, it is possible to examine the effects of for example, several ionic solutions and chemicals sequentially in the same preparation, and also to compare electric properties of different preparations. In the present study, we carried out MEA measurements in ileal musculatures of WT and *W/W^v^* mice. In the latter preparations, it is well known that the number of ICC, gut pacemaker cells, is reduced due to a loss-of-function mutation of c-Kit receptor gene [5], [7], [15]. In agreement with this genetic mutation, spatio-temporal analyses of the MEA data clearly distinguished these two preparations (Figs. 4–6).

In MEA measurements, nevertheless, several issues that can affect the quality of the study need to be considered. Firstly, if electric activity propagates only in the core of the tissue through intercellular coupling via gap junction channels, and regeneration of cellular electric activity (current) is negligible in the surface of the tissue {see Appendix in [30]}, extracellular electrodes in MEA do not detect changes in field potential. Such spontaneous electric activity may occur in some preparations like detrusor smooth muscle of the urinary bladder [31]. However, pacemaker electric activities are measurable in the stomach and small intestine [16], [25], indicating that these potentials reflect local ionic channel current near electrodes at least in these preparations.

Secondly, mechanical activity may affect measurements in normal solution. Such a possibility has been suggested in extracellular recordings of the murine stomach [17]. However, mechanical effects appear to be small in ileal musculature preparations used in the present study, because in contrast to *W/W^v^* mice, random distribution of phase-shift and poor cross-correlation were never observed in WT mice even after the application of nifedipine, where mechanical activity was negligible (Fig. 6D–F). Also, in principle, extracellular recordings detect changes in the field potential (*E*_f_) as a product of local membrane current (*I*_m_) and the resistance between extracellular electrodes and the reference electrode (referred to as access resistance, *R*_a_):
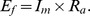



Therefore, it is deduced that oscillating field potentials imply the existence of oscillating membrane currents, and that mechanical activity alone does not induce any oscillations in *E*_f_ through changes in *R*_a_, if *I*_m_ = 0.

Thirdly, it is important to apply an appropriate frequency of high-pass filtering. The impedance of the recording microelectrode (*Z*_ME_) is given as a function of signal frequency (*f*):
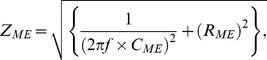
where *C*_ME_ and *R*_ME_ are the capacitance and resistance of the recording microelectrode, respectively. In recording slow electric oscillations such as in the gut, therefore, *C*_ME_ is required to increase in order to decrease the impedance, because the former component, capacitive reactance, of the function increases at a low frequency. For this reason, we have employed microelectrodes made of platinum black nano-particles increasing the surface area ∼200-folds (Fig. 1 and Fig. S1), being equivalent to ∼0.5 mm^2^ with a capacitance of 0.052 µF. By using recent nanofabrication techniques, e.g. nanosphere lithography [32], electrodes with increased surface area may be designed differently in the future. Such electrodes are also advantageous in reducing thermal noise due to lower resistance. On the other hand, if ordinary extracellular microelectrodes are used in MEA, it is difficult to measure slowly oscillating potentials due to the electrical impedance mismatch.

Early studies of ICC had been focused on how each of these special interstitial cells generates electric potentials periodically. Thus, it has become clearer that intracellular Ca^2+^ oscillations play a central role. Namely, Ca^2+^-activated ionic conductance in the plasma membrane, such as Ca^2+^-activated Cl^-^ channels and nonselective cation channels, are periodically activated to generate pacemaker current [9]. In the light of the gut function in which coordinated actions are required in smooth and elaborate motility, such as in peristalsis and segmentation movements, we should next consider how Ca^2+^ oscillations are linked between the pacemaking interstitial cells.

It is recognized that the ICC network as well as the enteric neuron network play a crucial role in coordinating gut motility. For example, the propagation of slow electric potentials is enhanced by distending the lumen in the small intestine [33], [34]. Also, there is an increasing body of evidence that the ICC network is impaired in diabetes mellitus frequently implicated by gut motility disorders [10,13]. Consistent with this notion, all three types of mapping analyses in the present MEA study, namely the use of spectral power, auto-correlation and cross-correlation parameters, showed a clear contrast between WT and *W/W^v^* mice, the latter being a model of an impaired ICC network. The random distributions of pseudo-colours in these maps are considered to be due to a reduced number of ICC and the resultant impairment of spatial electric connectivity.

Dihydropyridine Ca^2+^ antagonists, such as nifedipine, suppress smooth muscle activity, but preserve pacemaker activity in ICC [35]–[37]. Also, TTX selectively blocks voltage-gated Na^+^ channels to inhibit neural activity. In the present study, applications of nifedipine and TTX have prolonged the phase-shift along the oral-to-anal (longitudinal) plane in musculature preparations in WT mice (B vs E; C vs F in Fig. 6). This effect is ascribed mainly to the inhibitory effect of nifedipine on smooth muscle, because the application of TTX alone has little effect (unpublished observation). Therefore, it is suggested that the excitability of smooth muscle cells also plays a role in coupling and propagating spontaneous electric activity in the ICC network. Furthermore, the fact that applications of nifedipine and TTX reduce the spectral power much more in WT mice than in *W/W^v^* mice (Fig. 4E and F) supports the cooperation of ICC and other excitable cells in the spatial electric coupling.

Several computer models have so far been reported for ICC pacemaker activity [26], [38]–[43]. All models are based on intracellular Ca^2+^ oscillations, simulating previous observations with inhibitors for intracellular Ca^2+^ release channels and Ca^2+^-permeable channels across the plasma membrane. Among these models, Ca^2+^ phase wave models [26], [38], [43] can address the synchronization of Ca^2+^ oscillations in the ICC network. In Ca^2+^ phase wave models, voltage-sensitive Ca^2+^ release mechanisms, such as depolarization-mediated IP_3_ release, are used to interact Ca^2+^ oscillations in individual ICC, and depolarisations are conducted via gap junctions. In intact musculature preparations it is considered that ICC pacemaker current conducts toward smooth muscle cells via gap junctions. It is likely that L-type Ca^2+^ channels, a major inward current for depolarization in smooth muscle cells [14], make a contribution to the intercellular coupling between ICC (Fig. S2). For example, when nifedipine suppresses inward current through L-type Ca^2+^ channels in smooth muscle, instead more pacemaker current from ICC is used to charge the plasma membrane in smooth muscle cells, thereby reducing the intercellular coupling between ICC by their own pacemaker current. In addition, if some voltage-sensitive Ca^2+^ channels, e.g. T-type Ca^2+^ channels exist in ICC, albeit a minor conductance [37], [44] and/or in a subpopulations of ICC, the intercellular coupling is enhanced to propagate pacemaker potentials [42], [45]. These hypotheses agree well with the present MEA observation of the alteration in the phase-shift (Fig. 6B and E).

In conclusion, the arrayed microelectrodes with increased surface area successfully measure slowly oscillating electric activity in a small area (∼1 mm^2^) of ileal musculatures, due to the low impedance of microelectrodes (∼31 MΩ at 0.1 Hz). Mapping of spectral power, and auto-correlation and cross-correlation parameters further characterized the spatial properties of spontaneous electric activity in the ileum of wild-type (WT) and *W/W^v^* mice, in addition to previous studies [16], [37]. Also, the electric potentials generated by ICC appear to be coupled via their own network, and enhanced by smooth muscle excitability. We hope that with sufficient biophysical and technical consideration, MEA will be utilized in future studies of slowly oscillating bio-electric potentials in a wide range of tissues and organs including the gut, because ICC-like interstitial cells suspected of generating spontaneous electric activity are distributed all over the body, and can be induced from embryonic stem (ES) cells and induced pluripotent stem (iPS) cells [9], [44]–[50].

## Supporting Information

Figure S1 3D structure of a recording electrode of a 50 µm×50 µm square made with platinum black particles. A: A photo of a recording electrode with profiles along with 1A–1B and 2A–2B. The profiles were measured with a confocal laser microscope. B: A pseudo-colour 3D reconstruction. The z-axis is shown expanded.
(DOC)

Figure S2 A hypothetic scheme. The coupling of spontaneous electric activity in an ICC network is supported by the excitability of adjacent cells electrically connected via gap junctions. For example, voltage-gated L-type Ca^2+^ channels generate a major inward current during depolarization. Therefore, when L-type Ca^2+^ channels are suppressed in smooth muscle, more pacemaker current from ICC is required to charge the plasma membrane of smooth muscle cells. As a result, the intercellular coupling between ICC is reduced.
(DOC)

## References

[1] Furness J (2006) The enteric nervous system. 1st ed. Wiley-Blackwell, Inc. Malden, MA. pp1–288.

[2] Wood JD (2006) Integrative function of enteric nervous system. In Physiology of the Gastrointestinal Tract, 4th ed. Vol 2. (eds Barrett KE, Ghishan FK, Merchant JL, Said HM, Wood JD, Johnson LR), San Diego, USA: Academic Press, 665–684.

[3] Tomita T (1981) Electrical activity (spikes and slow waves) in gastrointestinal smooth muscle. In Smooth Muscle: An Assessment of Current Knowledge. Eds. Bülbring E, Brading AF, Jones AW, Tomita T. Edward Arnold, London. 127–156.

[4] Szurszewski JH (1987) Electrical basis for gastrointestinal motility. In Physiology of Gastrointestinal Tract, 2nd edition. ed. Johnson LR. Raven Press, New York. pp383–422.

[5] Maeda H, Yamagata A, Nishikawa S, Yoshinaga K. Kobayashi S, et al (1992) Requirement of c-kit for development of intestinal pacemaker system. Development 116: 369–375. 1283735 10.1242/dev.116.2.369

[6] Faussone-Pellegrini MS, Thuneberg L (1999) Guide to the identification of interstitial cells of Cajal. Microsc Res Tech. 47: 248–266. 10.1002/(SICI)1097-0029(19991115)47:4<248::AID-JEMT4>3.0.CO;2-W 10602286

[7] Sanders KM, Ordög T. Koh SD. Torihashi S, Ward SM (1999) Development and plasticity of interstitial cells of Cajal. Neurogastroenterol Motil. 11: 311–338. 10.1046/j.1365-2982.1999.00164.x 10520164

[8] Rumessen JJ, Vandervinden J–M (2003) Interstitial cells in the musculature of the gastrointestinal tract: Cajal and beyond. Int Rev Cytol. 229: 115–208. 10.1016/s0074-7696(03)29004-5 14669956

[9] Takaki M, Suzuki H, Nakayama S (2010) Recent advances in studies of spontaneous activity in smooth muscle: ubiquitous pacemaker cells. Prog Biophys Mol Biol. 102: 129–135. 10.1016/j.pbiomolbio.2010.05.007 20553741

[10] Camilleri M (2002) Advances in diabetic gastroparesis. Rev Gastroenterol Disord. 2: 47–56. 12122960

[11] Wang XY, Berezin I, Mikkelsen HB, Der T, Bercik P, et al (2002) Pathology of interstitial cells of Cajal in relation to inflammation revealed by ultrastructure but not immunohistochemistry. Am J Pathol. 160: 1529–1540. 10.1016/s0002-9440(10)62579-5 PMC1867230 11943737

[12] Kinoshita K, Horiguchi K, Fujisawa M, Kobirumaki F, Yamato S, et al (2007) Possible involvement of muscularis resident macrophages in impairment of interstitial cells of Cajal and myenteric nerve systems in rat models of TNBS-induced colitis. Histochem Cell Biol 127: 41–53. 16871386 10.1007/s00418-006-0223-0

[13] Vittal H, Farrugia G, Gomez G, Pasricha PJ (2007) Mechanisms of disease: the pathological basis of gastroparesis a review of experimental and clinical studies. Nat Clin Pract Gastroenterol Hepatol. 4: 336–346. 17541447 10.1038/ncpgasthep0838

[14] Akbarali HI, Hawkins EG, Ross GR, Kang M (2010) Ion channel remodeling in gastrointestinal inflammation. Neurogastroenterol Motil. 22: 1045–1055. 10.1111/j.1365-2982.2010.01560.x PMC2939949 20618833

[15] Iino S, Horiguchi S, Horiguchi K, Nojyo Y (2007) Interstitial cells of Cajal in the gastrointestinal musculature of W mutant mice. Arch Histol Cytol. 70: 163–173. 10.1679/aohc.70.163 18079585

[16] Nakayama S, Ohishi R, Sawamura K, Watanabe K, Hirose K (2009) Microelectrode array evaluation of gut pacemaker activity in wild-type and *W/W^v^* mice. Biosens Bioelectron. 25: 61–67. 10.1016/j.bios.2009.06.006 19576758

[17] Bayguinov O, Hennig GW, Sanders KM (2011) Movement based artifacts may contaminate extracellular electrical recordings from GI muscles. Neurogastroenterol Motil. 23: 1029–1042. 10.1111/j.1365-2982.2011.01784.x PMC4793914 21951699

[18] Rhee PL, Lee JY, Son HJ, Kim JJ, Rhee JC, et al (2011) Analysis of pacemaker activity in the human stomach. J Physiol. 589: 6105–6118. 10.1113/jphysiol.2011.217497 PMC3286689 22005683

[19] O’Grady G (2012) Gastrointestinal extracellular electrical recordings: fact or artifact? Neurogastroenterol Motil 24: 1–6. 10.1111/j.1365-2982.2011.01815.x PMC3245636 22188324

[20] O’Grady G, Pullan AJ, Cheng LK (2012) The analysis of human gastric pacemaker activity. J Physiol. 590: 1299–1300. 10.1113/jphysiol.2011.224014 PMC3381832 22399822

[21] Nakayama S (2012) Frequency analysis may distinguish the effects of calcium antagonists on mechanical and electrical activity. Neurogastroenterol Motil. 24: 397. 10.1111/j.1365-2982.2012.01882.x 22414186

[22] Oka H, Shimono K, Ogawa R, Sugihara H, Taketani M (1999) A new planar multielectrode array for extracellular recording: application to hippocampal acute slice. J Neurosci Methods. 93: 61–67. 10.1016/s0165-0270(99)00113-2 10598865

[23] Lammers WJ, Ver Donck L, Stephen B, Smets D, Schuurkes JA (2008) Focal activities and re-entrant propagations as mechanisms of gastric tachyarrhythmias. Gastroenterology. 135: 1601–1611. 10.1053/j.gastro.2008.07.020 18713627

[24] Du P, O’Grady G, Egbuji JU, Lammers WJ, Budgett D, et al (2009) High-resolution mapping of in vivo gastrointestinal slow wave activity using flexible printed circuit board electrodes: methodology and validation. Ann Biomed Eng. 37: 839–846. 10.1007/s10439-009-9654-9 PMC4090363 19224368

[25] Nakayama S, Shimono K, Liu HN, Jiko H, Katayama N, et al (2006) Pacemaker phase shift in the absence of neural activity in guinea-pig stomach: a microelectrode array study. J Physiol. 576: 727–738. 10.1113/jphysiol.2006.118893 PMC1890421 16990400

[26] van Helden DF, Imtiaz MS (2003) Ca^2+^ phase waves: a basis for cellular pacemaking and long-range synchronicity in the guinea-pig gastric pylorus. J Physiol. 548: 271–296. 10.1113/jphysiol.2002.033720 PMC2342787 12576498

[27] Hirst GD, Garcia-Londono AP, Edwards FR (2006) Propagation of slow waves in the guinea-pig gastric antrum. J Physiol. 571: 165–177. 10.1113/jphysiol.2005.100735 PMC1805648 16357017

[28] Tsutsui H, Higashijima S, Miyawaki A, Okamura Y (2010) Visualizing voltage dynamics in zebrafish heart. J Physiol. 588: 2017–2021. 10.1113/jphysiol.2010.189126 PMC2911208 20421282

[29] Vogt KE, Gerharz S, Graham J, Canepari M (2011) High-resolution simultaneous voltage and Ca^2+^ imaging. J Physiol. 589: 489–494. 10.1113/jphysiol.2010.200220 PMC3055538 21115640

[30] Nakayama S, Atsuta S, Shinmi T, Uchiyama T (2011) Pulse-driven magnetoimpedance sensor detection of biomagnetic fields in musculatures with spontaneous electric activity. Biosens Bioelectron. 27: 34–39. 10.1016/j.bios.2011.05.041 21741817

[31] Bramich NJ, Brading AF (1996) Electrical properties of smooth muscle in the guinea-pig urinary bladder. J Physiol. 492: 185–198. 10.1113/jphysiol.1996.sp021300 PMC1158872 8730594

[32] Yagati AK, Kim SU, Min J, Choi JW (2009) Multi-bit biomemory consisting of recombinant protein variants, azurin. Biosens Bioelectron 24: 1503–1507. 18809307 10.1016/j.bios.2008.07.080

[33] Huizinga JD, Ambrous K, Der-Silaphet T (1998) Co-operation between neural and myogenic mechanisms in the control of distension-induced peristalsis in the mouse small intestine. J Physiol. 506: 843–856. 10.1111/j.1469-7793.1998.843bv.x PMC2230746 9503342

[34] Seerden TC, Lammers WJ, De Winter BY, De Man JG, Pelckmans PA (2005) Spatiotemporal electrical and motility mapping of distension-induced propagating oscillations in the murine small intestine. Am J Physiol Gastrointest Liver Physiol. 289: G1043–1051. 10.1152/ajpgi.00205.2005 16099869

[35] Dickens EJ, Hirst GDS, Tomita T (1999) Identification of rhythmically active cells in guinea-pig stomach. J Physiol. 514: 515–531. 10.1111/j.1469-7793.1999.515ae.x PMC2269070 9852332

[36] Huang SM, Nakayama S, Iino S, Tomita T (1999) Voltage sensitivity of Slow wave frequency in isolated circular muscle strips from guinea pig gastric antrum. Am J Physiol Gstrointestinal Liver. 276: G518–528. 10.1152/ajpgi.1999.276.2.G518 9950827

[37] Kito Y, Ward SM, Sanders KM (2005) Pacemaker potentials generated by interstitial cells of Cajal in the murine intestine. Am J Physiol Cell Physiol. 288: C710–720. 10.1152/ajpcell.00361.2004 15537708

[38] Imtiaz MS, Katnik CP, Smith DW, van Helden DF (2006) Role of voltage-dependent modulation of store Ca^2+^ release in synchronization of Ca^2+^ oscillations. Biophys J. 90: 1–23. 10.1529/biophysj.104.058743 PMC1367009 16040741

[39] Youm JB, Kim N, Han J, Kim E, Joo H, et al (2006) A mathematical model of pacemaker activity recorded from mouse small intestine. Philos Transact A Math Phys Eng Sci. 364: 1135–1154. 10.1098/rsta.2006.1759 16608700

[40] Faville RA, Pullan AJ, Sanders KM, Koh SD, Lloyd CM, et al (2009) Biophysically based mathematical modeling of interstitial cells of Cajal slow wave activity generated from a discrete unitary potential basis. Biophys J. 96: 4834–4852. 10.1016/j.bpj.2009.03.058 PMC2712030 19527643

[41] Means SA, Sneyd J (2010) Spatio-temporal calcium dynamics in pacemaking units of the interstitial cells of Cajal. J Theor Biol. 267: 137–152. 10.1016/j.jtbi.2010.08.008 20705074

[42] Buist ML, Corrias A, Poh YC (2010) A model of slow wave propagation and entrainment along the stomach. Ann Biomed Eng. 38: 3022–3030. 10.1007/s10439-010-0051-1 20437204

[43] van Helden DF, Laver DR, Holdsworth J, Imtiaz MS (2010) Generation and propagation of gastric slow waves. Clin Exp Pharmacol Physiol 37: 516–524. 19930430 10.1111/j.1440-1681.2009.05331.x

[44] Hotta A, Kito Y, Suzuki H (2005) The effects of flufenamic acid on spontaneous activity of smooth muscle tissue isolated from the guinea-pig stomach antrum. J Smooth Muscle Res. 41: 207–220. 10.1540/jsmr.41.207 16258234

[45] O’Grady G, Du P, Paskaranandavadivel N, Angeli TR, Lammers WJ, et al (2012) Rapid high-amplitude circumferential slow wave propagation during normal gastric pacemaking and dysrhythmias. Neurogastroenterol Motil. 24: e299–e312. 10.1111/j.1365-2982.2012.01932.x PMC3383091 22709238

[46] Huizinga JD, Faussone-Pellegrini MS (2005) About the presence of interstitial cells of Cajal outside the musculature of the gastrointestinal tract. J Cell Mol Med. 9: 468–473. 10.1111/j.1582-4934.2005.tb00372.x PMC6740097 15963266

[47] Harhun MI, Pucovsky V, Povstyan OV, Gordienko DV, Bolton TB (2005) Interstitial cells in the vasculature. J Cell Mol Med. 9: 232–243. 10.1111/j.1582-4934.2005.tb00352.x PMC6740307 15963246

[48] Ishikawa T, Nakayama S, Nakagawa T, Horiguchi K, Misawa H, et al (2004) Characterization of in vitro gut-like organ formed from mouse embryonic stem cells. Am J Physiol Cell Physiol. 286: C1344–1352. 10.1152/ajpcell.00392.2003 14960414

[49] Brading AF, McCloskey KD (2005) Mechanisms of disease: specialized interstitial cells of the urinary tract-an assessment of current knowledge. Nat Clin Pract Urol. 2: 546–554. 10.1038/ncpuro0340 16474598

[50] Lang RJ, Tonta MA, Zoltkowski BZ, Meeker WF, Wendt I, et al (2006) Pyeloureteric peristalsis: role of atypical smooth muscle cells and interstitial cells of Cajal-like cells as pacemakers. J Physiol. 576: 695–705. 10.1113/jphysiol.2006.116855 PMC1890417 16945969

