# Supplementary material for: Spatial Analysis of Slowly Oscillating Electric Activity in the Gut of Mice Using Low Impedance Arrayed Microelectrodes
Source: PLoS One. 2013 Oct 4;8(10):e75235. doi: 10.1371/journal.pone.0075235 (PMC3790767; doi:10.1371/journal.pone.0075235)
Supplement: Figure S1 — 3D structure of a recording electrode of a 50 µm×50 µm square made with platinum black particles. A: A photo of a recording electrode with profiles along with 1A–1B and 2A–2B. The profiles were measured with a confocal laser microscope. B: A pseudo-colour 3D reconstruction. The z-axis is shown expanded. (DOC) [file pone.0075235.s001.doc]

**Supporting Information: Supplemental Figures 1.**


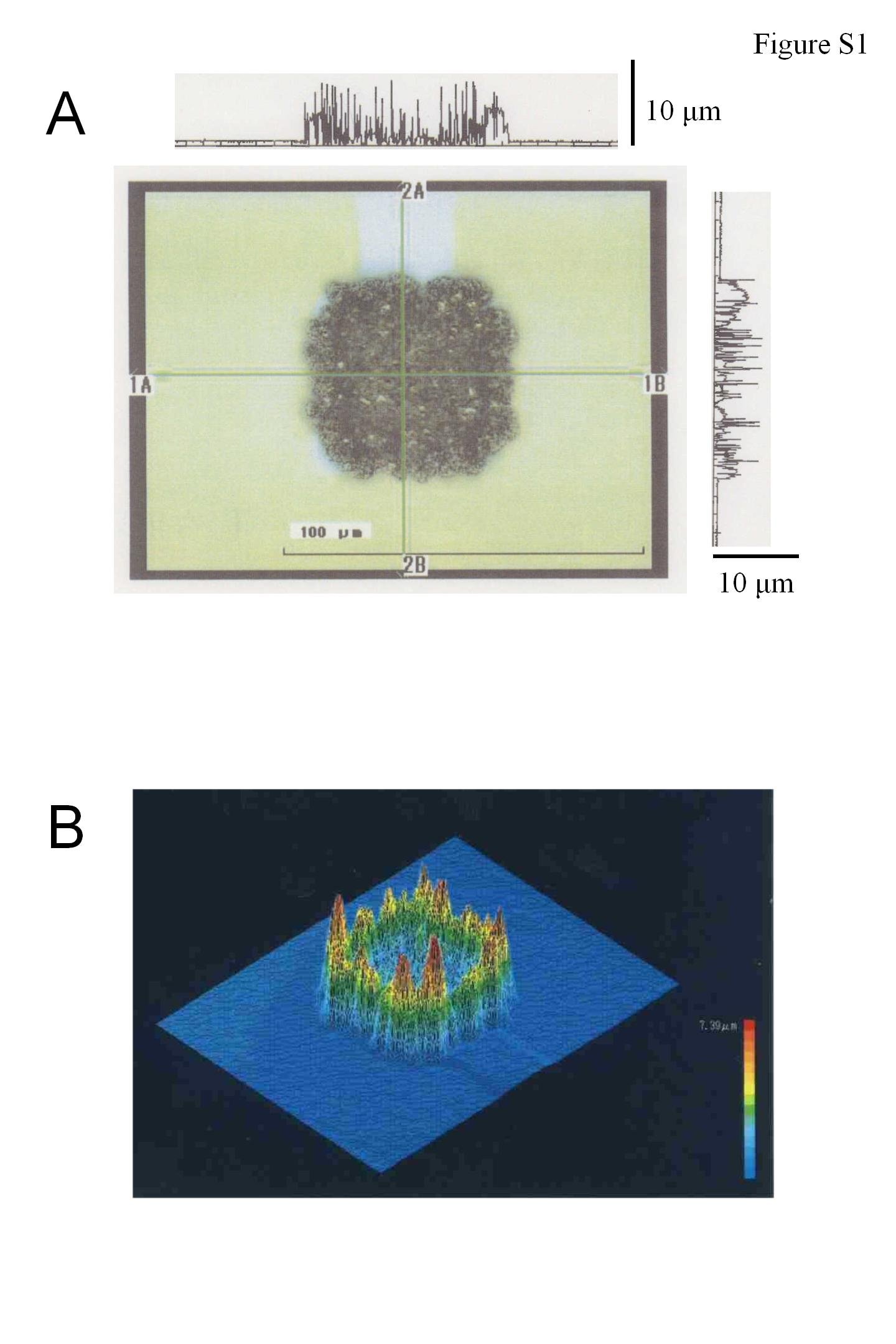


Figure S1. 3D structure of a recording electrode of a 50 μm × 50 μm square made with platinum black particles. A: A photo of a recording electrode with profiles along with 1A-1B and 2A-2B. The profiles were measured with a confocal laser microscope. B: A pseudo-colour 3D reconstruction. The z-axis is shown expanded.
