# Supplementary material for: Spatial Analysis of Slowly Oscillating Electric Activity in the Gut of Mice Using Low Impedance Arrayed Microelectrodes
Source: PLoS One. 2013 Oct 4;8(10):e75235. doi: 10.1371/journal.pone.0075235 (PMC3790767; doi:10.1371/journal.pone.0075235)
Supplement: Figure S2 — A hypothetic scheme. The coupling of spontaneous electric activity in an ICC network is supported by the excitability of adjacent cells electrically connected via gap junctions. For example, voltage-gated L-type Ca2+ channels generate a major inward current during depolarization. Therefore, when L-type Ca2+ channels are suppressed in smooth muscle, more pacemaker current from ICC is required to charge the plasma membrane of smooth muscle cells. As a result, the intercellular coupling between ICC is reduced. (DOC) [file pone.0075235.s002.doc]

**Supporting Information: Supplemental Figures 2.**


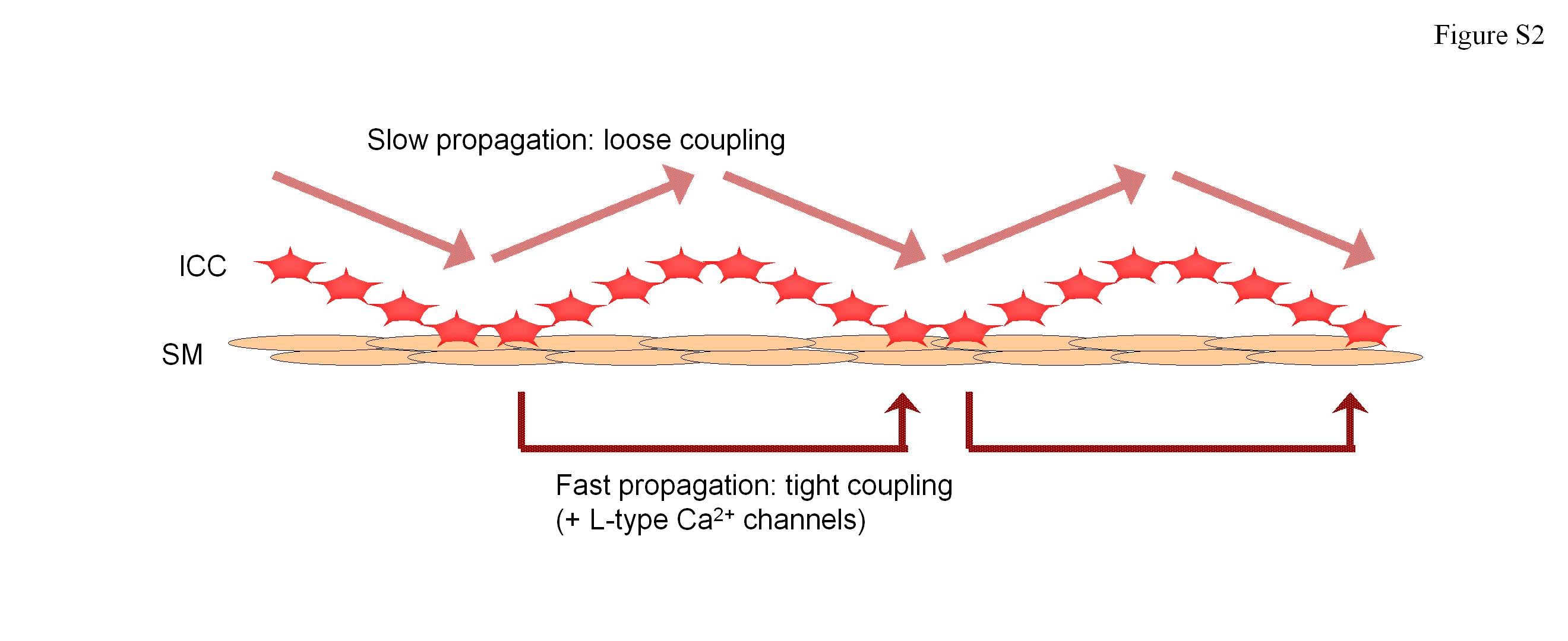


Figure S2. A hypothetic scheme. The coupling of spontaneous electric activity in an ICC network is supported by the excitability of adjacent cells electrically connected via gap junctions. For example, voltage-gated L-type Ca^2+^ channels generate a major inward current during depolarization. Therefore, when L-type Ca^2+^ channels are suppressed in smooth muscle, more pacemaker current from ICC is required to charge the plasma membrane of smooth muscle cells. As a result, the intercellular coupling between ICC is reduced.
